# A dens fracture case solved

**DOI:** 10.1093/rheumatology/keae026

**Published:** 2024-02-08

**Authors:** Ling Oei, Jiawei Li, A Faiz Karim, Robert M Verdijk, Edwin H G Oei, Jan A M van Laar, David Ten Cate, Iain Haitsma, Dominiek A Monserez, M Carola Zillikens

**Affiliations:** Department of Internal Medicine, Erasmus MC, University Medical Center, Rotterdam, The Netherlands; Department of Internal Medicine, Erasmus MC, University Medical Center, Rotterdam, The Netherlands; Department of Internal Medicine, Groene Hart Hospital, Gouda, The Netherlands; Department of Pathology, Erasmus MC, University Medical Center, Rotterdam, The Netherlands; Department of Radiology and Nuclear Medicine, Erasmus MC, University Medical Center, Rotterdam, The Netherlands; Department of Internal Medicine, Erasmus MC, University Medical Center, Rotterdam, The Netherlands; Department of Immunology, Erasmus MC, University Medical Center, Rotterdam, The Netherlands; Department of Rheumatology, Erasmus MC, University Medical Center, Rotterdam, The Netherlands; Department of Rheumatology, Sint Maartenskliniek, Nijmegen, The Netherlands; Department of Neurosurgery, Erasmus MC, University Medical Center, Rotterdam, The Netherlands; Department of Otolaryngology, Erasmus MC, University Medical Center, Rotterdam, The Netherlands; Department of Internal Medicine, Erasmus MC, University Medical Center, Rotterdam, The Netherlands

## Abstract

**Figure:**
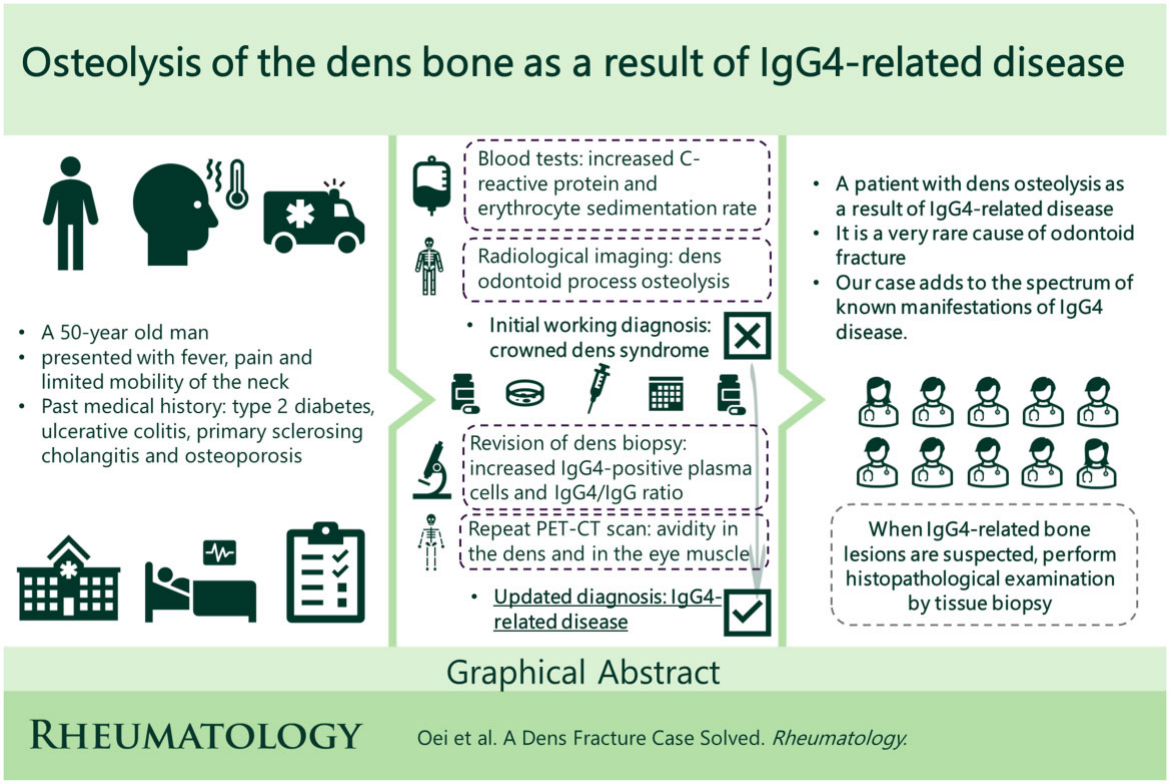

Rheumatology key message

- IgG4-RD should be included in the differential diagnosis of lytic bone lesions.

Dear Editor, IgG4-related disease (IgG4-RD) is a fibroinflammatory condition [1] characterized by dense lymphoplasmocytic infiltration rich in IgG4-positive plasma cells, storiform fibrosis and prompt response to corticosteroids. IgG4-RD commonly presents as salivary and lacrimal gland enlargement, orbital disease, autoimmune pancreatitis, retroperitoneal fibrosis and tubulointerstitial nephritis [2]. It sometimes mimics malignancy by inflammatory pseudotumors, complicating correct diagnosis and treatment. Rarely IgG4-RD causes lytic bone lesions, which is known for an aggressive course [3, 4]. Nonetheless, the involvement of the dens has not yet been reported. This case is the first report of osteolysis of the dens due to IgG4-RD, which should be added to the differential diagnosis of lytic bone lesions.

The patient was a 50-year-old male with a non-united dens fracture. He presented with sudden severe pain in the neck, limited and painful mobility of the neck and fever. Past medical history included type 2 diabetes, ulcerative colitis, primary sclerosing cholangitis and bisphosphonate use for 3 years for osteoporosis. He was not on immunosuppressive therapy. No focal neurologic deficits were found. CRP was 67 mg/l and ESR was 62 mm/h. Serum calcium, phosphorus, creatinine, anti-CCP, angiotensin-converting enzyme, 25-hydroxyvitamin D and 1,25-dihydroxyvitamin D were normal, alkaline phosphatase was slightly increased and IgG4 was within the reference range. Infectious meningitis was ruled out by lumbar puncture. Imaging showed calcification around the dens and inflammation of soft tissue. Therefore, a working diagnosis of crowned dens syndrome (CDS) was initially made. NSAID (celecoxib) and prednisolone treatments initially resulted in a biochemically good response. However, within weeks, destructive and expansive osteolysis of the dens and anterior arch of the atlas arose. Although inflammatory markers decreased, a non-united fracture remained (Fig. 1I).

**Figure 1.**
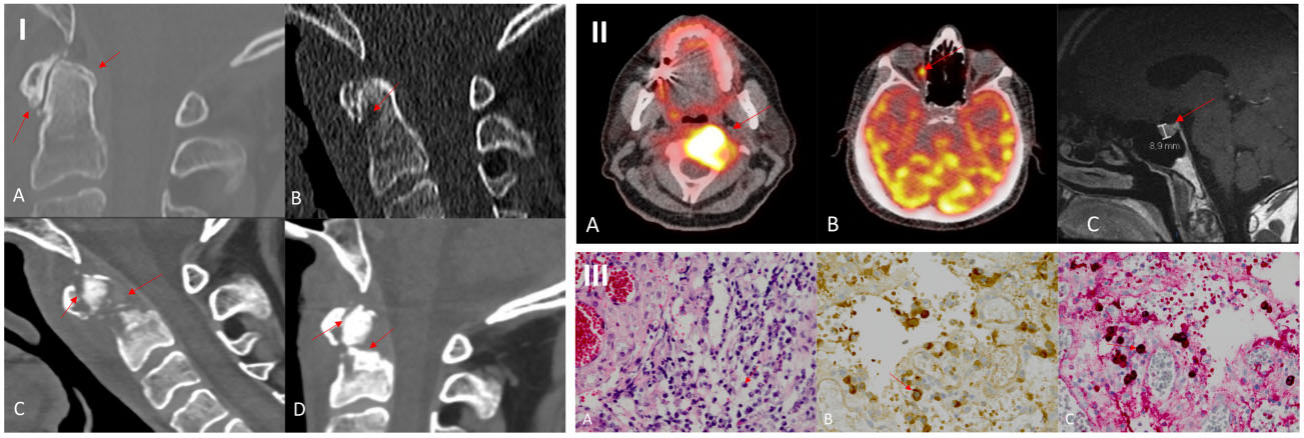
Imaging and histopathological results of the dens and surrounding tissues. **I.** CT scans of the dens. **(A)** Initial CT shows degenerative changes of the C1–C2 joint with small calcifications. **(B)** Low-dose CT shows lucency of the dens as a first sign of osteolysis. **(C)** Destructive and expansive osteolysis of the dens and anterior atlas arose. **(D)** With 2 months of prednisolone treatments, a radiological improvement with more ossification was seen. Yet the non-union fracture remained. **II.**  ^18^F-FDG PET/CT. **(A)**  ^18^F-FDG PET/CT without prednisolone treatment showed avid FDG uptake in and around the dens. **(B)** PET/CT without prednisolone additionally showed avidity in the medial rectus eye muscle. **(C)** MRI showed an enlargement of the pituitary. **III.** Histopathology results of the dens and surrounding tissues. **(A)** Haematoxylin and eosin staining showed scattered moderate infiltration of bone with lymphocytes and plasma cells. **(B)** IgG immunostaining showed IgG-positive plasma cells. **(C)** IgG4 immunostaining showed IgG4-positive plasma cells within the tissues

CDS was deemed unlikely, as there were no improvements of the neck symptoms and osteolysis after 2 months of prednisolone treatment. Also, the aggressive course did not fit and the radiologist had doubts about CDS because there were only some small calcifications and no typical ‘crown’ or ‘halo’ configuration. X-rays of the hand, knees and feet were also normal. Biopsy of the dens and surrounding soft tissues showed localized crystalline material, scattered moderate infiltration of bone with lymphocytes, plasma cells and focal crystalline birefringent material, but no clear calcium pyrophosphate crystals. Immunohistochemistry showed CD3-positive T cells and CD79a- and CD138-positive plasma cells (Fig. 1II) and no evidence of malignancy, lymphoma or multiple myeloma. ^18^F-fluorodeoxyglucose (^18^F-FDG) PET under prednisolone showed no abnormalities.

Despite normal serum IgG4, pathology revision demonstrated dense lymphocytic fibrosing infiltrate with >100 IgG4-positive plasma cells/HPF and an IgG4:IgG ratio of 50%, highly suspicious of IgG4-RD (Fig. 1II). Another ^18^F-FDG PET/CT without prednisolone showed increased radiotracer activity in the dens and additionally in the right medial rectus eye muscle, supporting the diagnosis of IgG4-RD (Fig. 1III). Further, an enlargement of the pituitary gland was found without PET avidity and was judged as an incidentally detected microadenoma. After multidisciplinary discussion, the working diagnosis of CDS was changed to IgG4-RD.

CDS, a condition in which the atlanto-occipital joint is affected by calcium pyrophosphate deposition disease [5], the second most common crystal-induced arthritis [6], presents with acute cervico-occipital pain and inflammation. Radiologically, CDS is characterized by peri-odontoid calcifications of the transverse ligament of the atlas.

In clinical practice, elevated serum IgG4 represents the only validated non-invasive biomarker for IgG4-RD. The association of serum IgG4 with diagnosis, prognosis, response to treatment and disease activity of IgG4-RD has been investigated. However, serum IgG4 is normal in approximately half of the patients with IgG4-RD [2].

Imaging may contribute to the diagnosis by means of ^18^F-FDG PET, which shows increased FDG uptake in the involved tissues. On MRI, homogeneous enhancement after gadolinium contrast administration also suggests the existence of IgG4-RD. However, some cases did not show enhancement after gadolinium on MRI and showed no enhancement on PET/CT, probably due to relatively low disease activity under use of immunosuppressors [3]. Most importantly, osteolytic lesions may not emerge during the early stages of IgG4-RD, as was the case in our patient. Once impairment of the odontoid process occurs, reversal by medical therapy is difficult.

To diagnose IgG4-RD, histopathology remains the gold standard and requires careful examination of the tissue, including immunohistochemical analysis with anti-IgG4 and anti-IgG antibodies. We recommend histopathological examination by tissue biopsy when IgG4-related bone lesions are highly suspected.

Our patient was followed up with repeat imaging and laboratory investigations by our multidisciplinary team. He received a neck collar for 8 months in total as a conservative treatment and he has been doing quite well except for persisting limited range of motion of the neck and neck pain. No progression or relapse of IgG4-RD has been observed to date. Our case adds to the spectrum of known manifestations of IgG4-RD.

## Data Availability

The data that has been used is confidential.
